# Endoscopic retrograde appendicitis therapy versus laparoscopic appendectomy versus open appendectomy for acute appendicitis: a pilot study

**DOI:** 10.1186/s12876-022-02139-7

**Published:** 2022-02-13

**Authors:** Zhemin Shen, Peilong Sun, Miao Jiang, Zili Zhen, Jingtian Liu, Mu Ye, Weida Huang

**Affiliations:** 1 Department of General Surgery, Jinshan Hospital, Fudan University, Shanghai, China; 2 Department of Gastroenterology, Jinshan Hospital, Fudan University, Shanghai, China

**Keywords:** Acute appendicitis, Endoscopic retrograde appendicitis therapy, Appendectomy, Randomized controlled trial

## Abstract

**Background:**

An increasing number of studies have shown the merits of endoscopic retrograde appendicitis therapy (ERAT) in diagnosing and treating acute uncomplicated appendicitis. However, no related prospective controlled studies have been reported yet. Our aim is to assess the feasibility and safety of ERAT in the treatment of acute uncomplicated appendicitis.

**Methods:**

In this open-label, randomized trial, participants were randomly allocated to the ERAT group, laparoscopic appendectomy (LA) group and open appendectomy (OA) group. The primary outcome was the clinical success rate of the treatment. Intention-to-treat analysis was used in the study.

**Results:**

The study comprised of 99 patients, with 33 participants in each group. The clinical success rate was 87.88% (29/33), 96.97% (32/33) and 100% (33/33) in the ERAT, LA and OA group, respectively. In the ERAT group, 4 patients failed ERAT due to difficult cannulation. In LA group, 1 patient failed because of abdominal adhesion. There were no significant differences among the three treatment groups regarding the clinical success rate (*P* = 0.123). The median duration of follow-up was 22 months. There were no significant differences (*P* = 0.693) among the three groups in terms of adverse events and the final crossover rate of ERAT to surgery was 21.21% (7/33).

**Conclusion:**

ERAT can serve as an alternative and efficient method to treat acute uncomplicated appendicitis.

*Trial registration* The study is registered with the WHO Primary Registry-Chinese Clinical Trial Registry (ChiCTR1900025812).

## Introduction

Acute appendicitis is one of the most common causes of acute abdominal pain clinically [1]. Appendectomy has long been standard treatment for acute appendicitis. However, there are a series of potential postoperative complications, such as postoperative bleeding, wound infection and intestinal obstruction [2, 3], and the overall complication rate has been reported to be 8.2–31.4% [1]. Moreover, negative appendectomy is also a nonnegligible problem [4]. As previous studies suggested that perforation may not be an inevitable consequence of acute appendicitis, there is a division of opinions on performing surgery on patients with acute uncomplicated appendicitis [5]. Thus, developing a safe and efficient nonoperative method has been an agenda for treating acute uncomplicated appendicitis.

Endoscopic retrograde appendicitis therapy (ERAT) was firstly reported by Liu et al. as being inspired by endoscopic retrograde cholangiopancreatography (ERCP) [6]. ERAT is a novel, nonoperative and minimally invasive method of treating acute uncomplicated appendicitis. Recently, there has been additional studies of the use of ERAT for treating acute uncomplicated appendicitis [7–9]. The results of these 3 trials indicated the clinical value of ERAT, including both diagnostic and therapeutic aspects. Thus, ERAT has the potential to become an alternative treatment method for acute appendicitis, especially in patients who are deemed as high-risk candidates for surgery. However, these previous studies were all retrospective, and no prospective study has been reported yet. To address this issue, we conducted a prospective randomized controlled trial to compare ERAT with laparoscopic appendectomy (LA) and open appendectomy (OA), and evaluated the feasibility and safety of ERAT in treating acute uncomplicated appendicitis.

## Method

### Patients

The period of patient enrollment was between January 2018 and August 2019. A prospective, open-label, randomized controlled study was conducted at Jinshan Hospital, Fudan University. Patients diagnosed with acute uncomplicated appendicitis were enrolled in the study.

The inclusion criteria were as follows: (1) patient age over 18 years and under 80 years; (2) Alvarado score > 5 [1]; (3) suspicious (or could not be excluded) acute appendicitis diagnosed by an abdominal CT scan, which was indicated by a dilated appendix with a diameter greater than 6 mm, a thickened cecal wall, and periappendiceal fat inflammation, with or without an appendicolith [10].

Exclusion criteria were as follows: (1) suspected acute complicated appendicitis with perforation or gangrene; (2) appendiceal diameter greater than 15 mm, which usually indicates malignancy [11]; (3) patients under the age of 18 years or over 80 years; (4) patients with the following contradictions for receiving colonoscopy, surgery or anesthesia: (a) severe cardiopulmonary insufficiency, psychiatric dysfunction or coma; (b) acute diffuse peritonitis, which is defined as diffuse abdominal tenderness, rebound tenderness and muscular tension; (c) acute gastroenteritis (dysentery, explosive ulcerative colitis); (d) concurrent menses; (e) intestinal obstruction; (f) acute gastrointestinal hemorrhage; (g) recent gastrointestinal or pelvic operation or radiotherapy; (h) allergy to contrast medium; (i) hemorrhagic tendency because of long-term use of corticosteroids or anticoagulant treatment; (5) patients undergoing any other clinical trial.

Written informed consent was obtained from all participants. The study was conducted according to the Declaration of Helsinki and approved by the Ethics Committee of Jinshan Hospital, Fudan University (No. 2017–24) on May 17th, 2017. The study is registered with the WHO Primary Registry-Chinese Clinical Trial Registry (ChiCTR1900025812) (09/09/2019).

Randomization was conducted by a computer-generated randomization number (1:1:1) by statisticians. The final allocation was concealed in an opaque envelope. The allocation was reported to the doctors and patients immediately prior to the intervention.

### Preintervention preparation

All patients intravenously received antibiotic treatment (1.5 g cefuroxime with 100 ml normal saline, 0.5% metronidazole 100 ml) immediately after being clinically diagnosed. In the ERAT group, the patients orally took 328.8 g polyethylene glycol (PEG) electrolyte solution with 2000 ml water before ERAT for bowel preparation. When the excrement became a clear liquid, the patients were well prepared to undergo ERAT.

### ERAT procedure

**Fig. 1:**
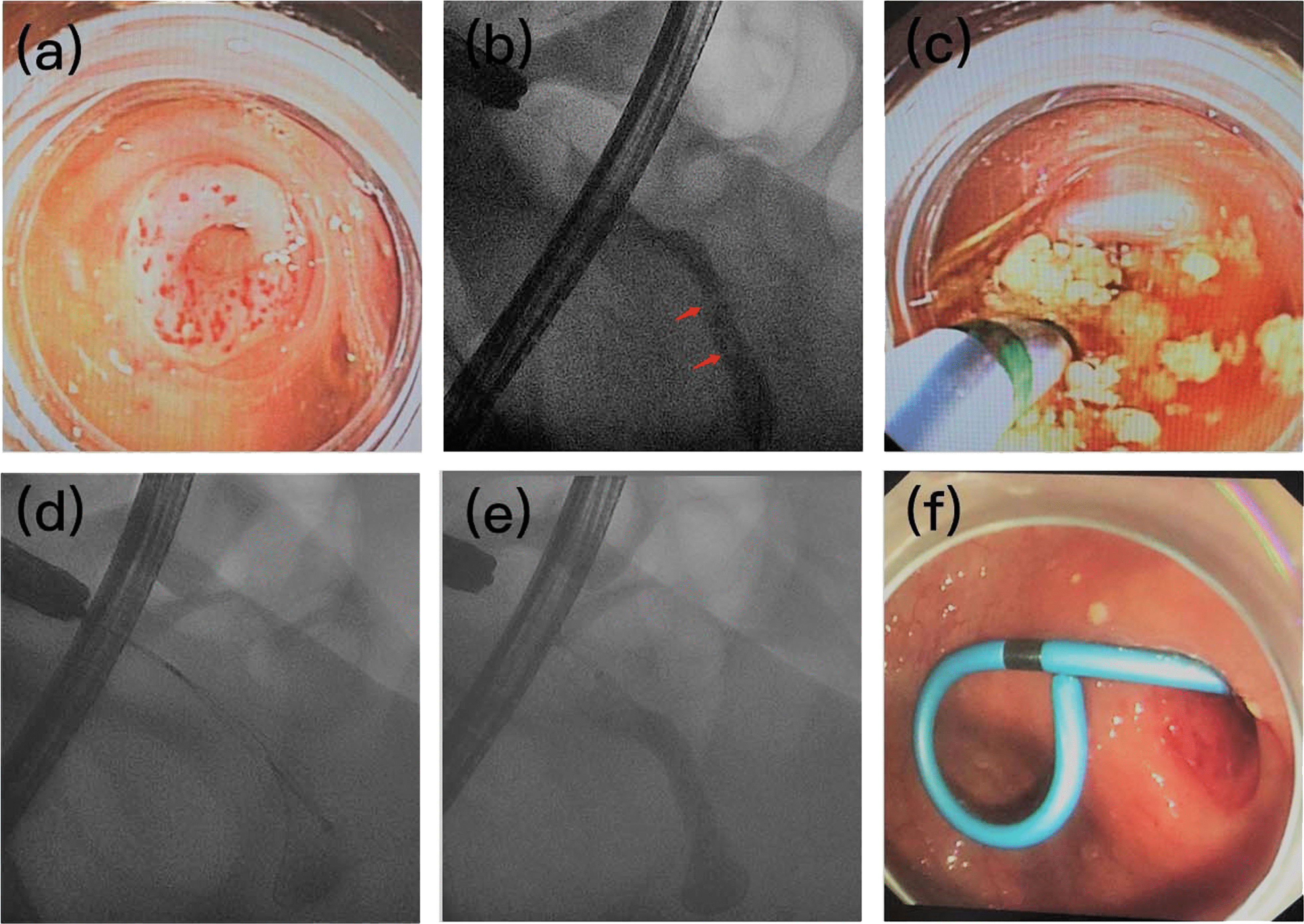
Procedures of endoscopic retrograde appendicitis therapy (ERAT). **a** Congestion and edema around the mucosa of appendix orifice. **b** The endoscopic retrograde appendicography (ERA) fluoroscopy showed filling defect in the appendix lumen (arrows), which indicated the presence of appendicoliths. **c** Cannulation of catheter along the guidewire with sand-like appendicoliths excretion and pus drainage inside the appendix lumen, confirming acute appendicitis. **d** Retracting the appendicoliths by the extraction basket. **e** After appendicolith being retracted, the appendix lumen was fully filled with contrast media under ERA. **f** Stenting for keeping pus drainage

Similar to that in previous studies [7–9], the ERAT procedure was performed as follows (Fig. 1):

First, a full and careful examination of the large intestine was performed by a colonoscope (CF-H260AI, Olympus, Japan) with a transparent cap. Then, the colonoscope was located to the appendiceal orifice to check the appendiceal mucosa to determine whether there was inflammation or any other abnormalities. With the help of a transparent cap, the tip of the catheter (BDC-12/55–7/1810/55–7/18, Micro-Tech Co. Ltd., Nanjing, China) was placed in the appendiceal orifice, and a 0.035-inch guidewire (MTN-BM-89/45-A, Micro-Tech Co. Ltd., Nanjing, China) was gently and deeply inserted into the appendiceal lumen over the catheter. Finally, the catheter moved forward into the appendiceal lumen along the guidewire. To make a definite diagnosis, the appendiceal lumen was filled with contrast medium (ioversol) for endoscopic retrograde appendicography (ERA) fluoroscopy. Next, the appendiceal lumen was flushed repeatedly with gentamicin (240,000 units with 100 ml normal saline) and 0.5% metronidazole 100 ml to clear pus and other infectious contents, such as sand-like appendicoliths. Then, an extraction basket (SEB-A- 30/55–7/200, Micro-Tech Co., Ltd., Nanjing, China) was used to extract the remaining appendicoliths if necessary. Finally, a plastic stent (SPSOF7-7, Cook, USA) was placed routinely in the appendiceal orifice to maintain pus drainage.

### Surgical treatment

All operations were performed by surgeons from a same team. In the LA group, the three-port technique (umbilical, 10 mm; suprapubic, 5 mm; right lower abdomen, 10 mm) was chosen. In the OA group, a McBurney muscle-splitting incision technique was used.

### Postintervention management

After ERAT or surgery was performed, patients were sent back to the ward and monitored carefully. Patients continued to receive anti-inflammatory therapy when necessary. In the ERAT group, patients were given a soft diet later on the same day and resumed a normal diet when the soft diet was tolerated. In the LA and OA groups, a soft diet was started 24 h after surgery, and a normal diet was given when the soft diet was completely tolerated. All patients were asked about their clinical presentation every day. Routine blood tests, including the white blood cell (WBC) count, neutrophil percentages and C-reactive protein (CRP) level, were performed on day 1, 3, and 5 in a similar fashion after ERAT or surgery. Patients were discharged when all symptoms were completely relieved and inflammatory markers (including the WBC count, neutrophil percentages, and CRP level) returned to normal. If ERAT failed or abdominal pain persisted after ERAT, LA or OA was performed immediately.

### Follow-up

Fourteen days after ERAT, all patients in the ERAT group were scheduled for outpatient services to inform doctors of their symptoms after discharge. Patients then received an abdominal X-ray to check the status of the stent. If the stent was not discharged spontaneously with defecation, colonoscopy was recommended to retrieve the stent.

Follow-up was performed by telephone interview every 3 months for the first half year, and then every 6 months till November 2020. In the ERAT group, recurrence of abdominal pain or appendicitis after ERAT, including the relevant treatment, was mainly investigated. In the LA group and OA group, the survey mainly included postoperative complications such as wound infection, persistent incision pain and intestinal obstruction. Follow-up was performed until the end of the study period.

### Outcomes

The primary outcome was the clinical success of the treatment. The secondary outcomes were as follows: the duration of complete relief of abdominal pain and body temperature; the duration of normalization of inflammatory markers including the WBC count; neutrophil percentages and CRP level; the duration of diet resumption; the length of hospital stay (LOS); the total cost of the primary hospital stay; adverse events during follow-up period and final crossover rate of ERAT to surgery.

#### ***Definition***

The clinical success of ERAT is defined as successful appendix cannulation with complete resolution of symptoms and normalization of inflammatory markers, including WBC count, neutrophil percentages and CRP. Difficult appendix cannulation is defined as failure to achieve successful appendix cannulation within 15 min. The assessment of abdominal pain degree is based on visual analog scales (VAS). In the VAS, scores of 0 and 10 represent no pain and most severe pain, respectively. Complete relief of abdominal pain refers to a VAS score of 0.

The diagnostic criteria of acute appendicitis by ERAT mainly consist of ERA fluoroscopy images, inflammation of the appendiceal mucosa observed under endoscopy and the presence of pus or appendicoliths inside the appendiceal lumen [8].

In the ERAT group, adverse events mainly indicated the recurrence of acute appendicitis. In the LA and OA groups, adverse events suggested postoperative complications.

### Statistical analysis

As this was a pilot trial, we did not perform a power calculation. On the basis of our yearly caseload of approximately 240 cases and estimated recruitment of one fifth of eligible cases, we aimed to enroll at least 96 patients within a 2-year period. Qualitative data are expressed as numbers (n) and percentages (%) and were compared by using the χ^2^ test or Fisher’s exact test when appropriate.

Quantitative data are expressed as the mean ± standard deviation (SD) or median with 25th and 75th percentiles, as appropriate. For normally distributed quantitative data (such as age, temperature, WBC count), one-way analysis of variance (ANOVA) was used to compare the differences among the three groups. For nonnormally distributed quantitative data (neutrophil percentages, CRP, Alvarado scores, VAS scores, the duration of normalization of inflammatory markers, the duration of normal diet and body temperature, total cost and length of hospitalization), the Kruskal–Wallis test followed by all pairwise multiple comparisons was used to detect statistical significance. Bonferroni’s correction was used for multiple hypothesis testing. The data were analyzed by IBM SPSS software version 22 (SPSS, Chicago, Illinois, USA). A *P* value < 0.05 was considered to be statistically significant. Intention-to-treat (ITT) analysis was performed after final allocation.

## Results

### Patients characteristics

**Fig. 2:**
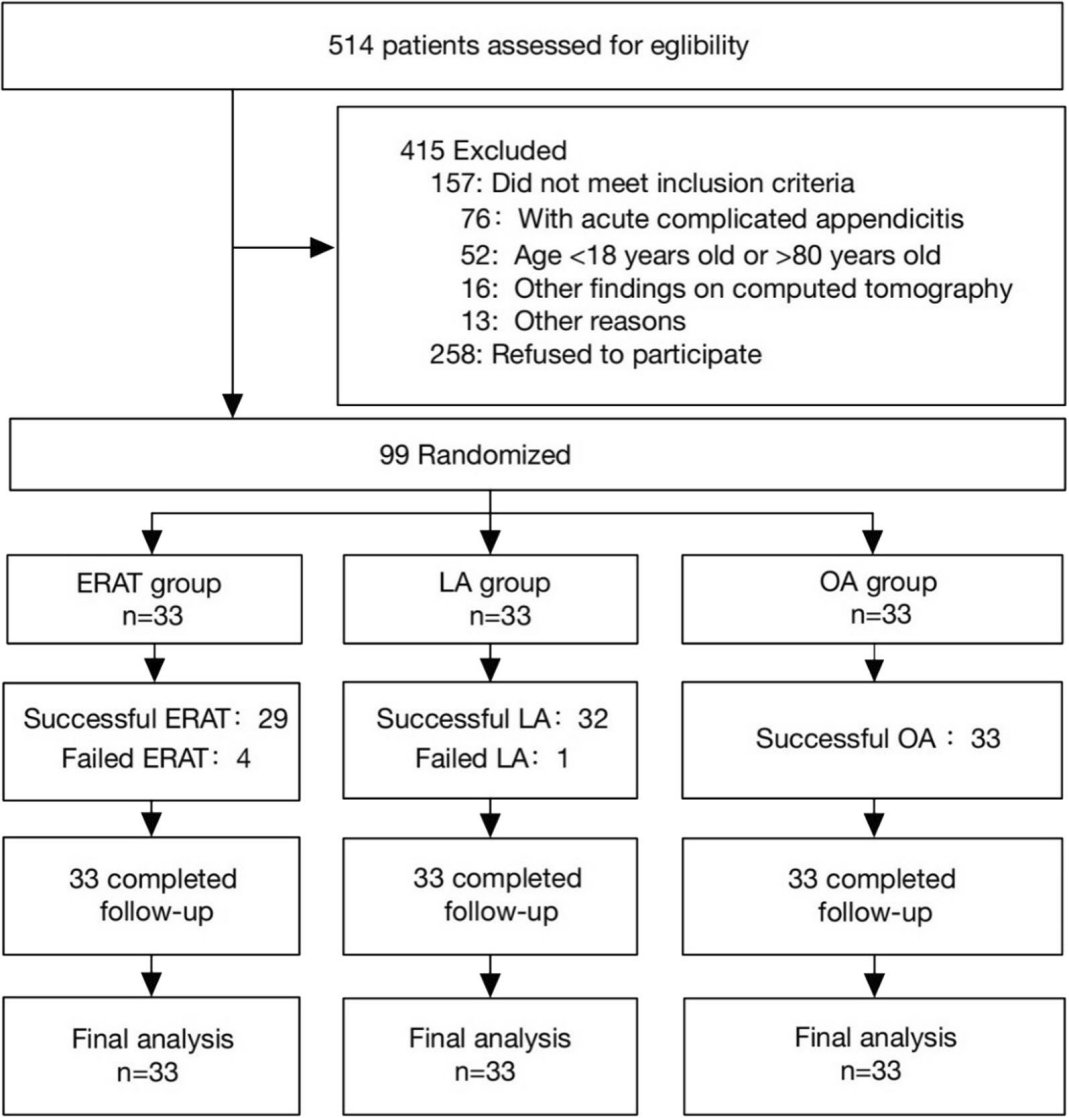
Trial profile

A total of 514 patients were assessed for eligibility, and 99 patients were enrolled in the study. There were 33 patients each in the ERAT group, LA group and OA group (Fig. 2). All 99 patients were finally analyzed. As indicated in Table 1, there were no significant differences observed in age, sex, clinical symptoms, signs, laboratory tests or abdominal CT scans. No signs of acute complicated appendicitis were observed via physical examination or abdominal CT scan among the 99 patients. Appendicoliths were present in 12 patients (36.36%) in the ERAT group, 16 patients (48.48%) in the LA group and 11 patients (33.33%) in the OA group. There were no significant differences in appendicoliths among the three groups (*p* = 0.516).

**Table 1:** Basic characteristics

	ERAT group (n = 33)	LA group (n = 33)	OA group (n = 33)	*P* value
Age, mean (SD), years	44.12 ± 16.95	43.24 ± 15.41	45.45 ± 18.04	0.866
Male, n (%)	18 (54.55)	17 (51.52)	19 (57.58)	0.967
Abdominal surgery history, n (%)	8 (24.24)	8 (24.24)	7 (21.21)	> 0.999
Symptoms				
Migrated right lower abdominal pain, n (%)	29 (87.88)	22 (66.67)	25 (75.76)	0.142
Nausea, n (%)	22 (66.67)	23 (69.70)	17 (51.52)	0.285
Vomiting, n (%)	5 (15.15)	4 (12.12)	9 (27.27)	0.345
Anorexia, n (%)	33 (100)	32 (96.97)	32 (96.97)	> 0.999
Fever, n (%)	18 (54.55)	13 (39.39)	17 (51.52)	0.532
Temperature (℃)	37.37 ± 0.77	37.13 ± 0.57	37.22 ± 0.58	0.317
Signs				
Right lower abdominal tenderness, n (%)	33 (100)	33 (100)	33 (100)	
Rebound tenderness, n (%)	17 (51.52)	15 (45.45)	20 (57.58)	0.654
Laboratory examination				
WBC count (× 10^9^/L)	11.78 ± 3.84	11.87 ± 3.80	13.64 ± 3.87	0.091
Neutrophil percentages, median (25th, 75th percentile)	0.84 (0.78, 0.87)	0.81 (0.76,0.85)	0.83 (0.76, 0.90)	0.233
CRP (mg/L)				0.749
0–20	18 (54.6)	20 (60.6)	25 (75.76)	
21–50	7 (21.2)	7 (21.2)	6 (18.18)	
> 50	8 (24.2)	6 (18.2)	2 (6.06)	
CT scan				
Local Cecal Wall thickening, n (%)	4 (12.12)	0 (0)	1 (3.03)	0.123
Periappendiceal fat inflammation, n (%)	25 (75.76)	27 (81.82)	29 (87.88)	0.496
Intraluminal appendicolith, n (%)	12 (36.36)	16 (48.48)	11 (33.33)	0.516
Alvarado scores, median (25th, 75th percentile)	8 (7, 9)	8 (6, 9)	8 (7, 9)	0.127
VAS scores, median (25th,75th percentile)	6 (5, 7)	7 (6, 7)	6 (6, 7)	0.054

ERAT, endoscopic retrograde appendicitis therapy; LA, laparoscopic appendectomy; OA, open appendectomy; SD, standard deviation; WBC, white blood cell; CRP, C-reactive protein; VAS, visual analog scale

### Treatment success rate and efficacy of ERAT

Table 2 shows the clinical outcomes of the three groups. Successful treatment was achieved in 29 patients (87.88%) in the ERAT group, 32 patients (96.97%) in the LA group and 33 patients (100%) in the OA group. No significant differences were observed among the three groups (*P* = 0.123). Among the three groups, ERAT failed in four patients (12.12%) due to difficult cannulation, and LA was performed in these patients later on the same day, with uneventful recoveries. All these four patients were found appendicoliths before ERAT. Appendicoliths of the other 8 patients who were found to have appendicoliths by CT scan were removed by ERAT. Stents were placed in 29 patients with successful ERAT. In the LA group, a 63-year-old patient failed LA due to extensive abdominal adhesion caused by surgical reduction for intussusception 3 years ago. The patient was later converted to OA successfully.

**Table 2:** Clinical outcome

	ERAT group (n = 33)	LA group (n = 33)	OA group (n = 33)	*P* value	ERAT-LA	ERAT-OA	LA-OA
Treatment Success, n (%)	29 (87.88)	32 (96.97)	33 (100)	0.123			
Definite acute uncomplicated appendicitis, n (%)	33 (100)	33 (100)	33 (100)				
Appendicoliths removal, n (%)	8 (66.67)	NA	NA				
Stent d rainage, n(%)	29 (87.88)	NA	NA				
Failed cannulation, n (%)	4 (12.12)	NA	NA				
Transferred to surgery, n (%)	4 (12.12)	NA	NA				
Normalization of WBC count, days				0.351			
0–1	16	12	17				
1–3	5	12	9				
> 3	2	0	2				
Normalization neutrophil percentages, days				0.607			
0–1	15	11	7				
1–3	11	14	19				
> 3	4	2	2				
Normalization of CRP, days				0.147			
0–1	3	1	3				
1–3	5	10	2				
> 3	7	2	3				
Normalization of temperature, median (25th, 75th percentile), days	2 (1, 3)	1 (1, 2)	2 (1, 3)	0.194			
LOS, median (25th, 75th percentile), days	5 (4, 6)	4 (3, 5)	4 (3, 5)	0.054			
Total cost, median (25th, 75th percentile), RMB	10,983.25 (10,210.26, 13,028.25)	14,517.22 (12,991.05, 15,528.25)	8246.82 (6819.71, 9530.22)	< 0.001	0.001	< 0.001	< 0.001

ERAT, endoscopic retrograde appendicitis therapy; LA, laparoscopic appendectomy; OA, open appendectomy; NA, not applicable; WBC, white blood cell; CRP, C-reactive protein; LOS, length of hospital stay

Among 29 patients who underwent successful ERAT, most achieved complete abdominal pain relief immediately after the procedure. The duration of normalization of inflammatory markers, including the WBC count, neutrophil percentages and CRP level, did not significantly differ among the ERAT, LA and OA groups (*P* = 0.351, *P* = 0.607, *P* = 0.147, respectively). The overall cost in the LA group was significantly higher (*P* < 0.001) than that in the ERAT group(including the second colonscopy to retrieve the stents) and OA group and was significantly lower (*P* < 0.001) in the OA group than in the ERAT group. Moreover, no significant differences (*P* = 0.054) were found in the LOS among the three groups.

### Follow-up

By the end of November 2020, all 99 patients were under follow-up. The median follow-up period was 22 months (25th and 75th percentiles: 19 months and 26 months). In the first 14 days after ERAT treatment, 20 patients (31.03%) received colonoscopy again to retrieve stents, while the stents of the other 9 patients (68.97%) were spontaneously discharged during the 14 days. Thus, among patients who underwent ERAT successfully, 20 patients received colonoscopy for two times and 9 patients only received colonoscopy for one time. Moreover, for the 4 patients who failed ERAT, they also received two-times intervention since they underwent both ERAT and LA. Table 3 summarizes the adverse events which arose in the three groups. No significant differences (*P* = 0.693) were found among the three treatment groups. In the ERAT group, three patients (9.09%) reported right lower abdominal pain and were diagnosed with recurrent acute appendicitis. The recurrence time was 4, 6 and 11 months, respectively. Two of them were found to have intraluminal appendicoliths during primary hospitalization. These three patients received LA later at the same day. Thus, there were totally 7 patients in the ERAT group who crossed over to the LA group, and the final crossover rate is 21.21% (7/33). In the LA group, one patient (3.03%) was found to have a fluid collection around the ileocecal junction and the fluid collection gradually diminished later on. In the OA group, three patients (9.09%) were found to have postoperative complications. Two of them exhibited wound infections within one month after surgery. Another patient complained of occasional incision pain one month after OA, especially on rainy days.

**Table 3:** Follow-up

	ERAT group (n = 33)	LA group (n = 33)	OA group (n = 33)	*P* value
Stent discharged, n (%)	29 (87.88)	NA	NA	
Spontaneously discharged, n (%)	9 (31.03)	NA	NA	
Retrieved, n (%)	20 (68.97)	NA	NA	
Adverse events, n (%)	3 (9.09)	1 (3.03)	3 (9.09)	0.693
Recurrence, n (%)	3 (9.09)	NA	NA	
Patients with appendicolith at first admission, n (%)	2 (6.06)	NA	NA	
Patients without appendicolith at first admission, n (%)	1 (3.03)	NA	NA	
Crossover rate (%)	21.21	3.03	NA	
Complications				
Wound infection, n (%)	NA	0	2 (6.06)	
Abdominal or incisional pain, n (%)	NA	0	1 (3.03)	
Abdominal fluid collection, n (%)	NA	1 (3.03)	0	
Obstructive symptoms, n (%)	NA	0	0	

ERAT, endoscopic retrograde appendicitis therapy; LA, laparoscopic appendectomy; OA, open appendectomy; NA, not applicable

## Discussion

In this pilot RCT, we have shown that ERAT is feasible and safe for treating acute uncomplicated appendicitis. Overall, nearly 25% of patients accepted to participate the trial, suggesting the potential clinical extension of ERAT since it is a brand-new treating method and no patient has ever heard of it before. Appendicoliths and infection are considered as important causes of acute appendicitis and ERAT could treat acute uncomplicated appendicitis by appendicolith extraction, pus drainage and intraluminal pressure reduction to relieve the syndromes and ultimately treat acute appendicitis [7, 12, 13]. Although this pilot trial was not adequately powered to detect differences in treatment efficacy, the results are useful to inform future clinical application of ERAT. Although there are some complications associated to colonoscopy, the adverse event rates were less than 1% and 0.2% for bleeding and perforation respectively and there were no any adverse events related to colonoscopy among the patients underwent ERAT treatment in our study [14]. Moreover, ERAT compares favorably with the results of surgery in terms of the postintervention recovery of patients. Regarding the period of body temperature normalization, inflammatory markers (including the WBC count, neutrophil percentages and CRP level) and the length of hospitalization, our data did not indicate significant differences among the three groups (*P* = 0.194, *P* = 0.351, *P* = 0.607, *P* = 0.147, *P* = 0.054, respectively), which showed that the clinical efficacy of ERAT was similar to that of surgery. As for different surgical methods, LA showed a similar clinical efficacy as OA, while LA yielded fewer complications, although there was no statistical significance (*P* = 0.693) in the current study. OA, as the traditional treatment method, is the most reliable therapy for acute appendicitis. However, OA seems to cause more adverse events, such as wound infection, which reduces the quality of life of patients.

A previous study suggested that the appendix could preserve and protect beneficial or commensal microorganisms from contamination with pathogenic organisms [15]. In addition, evidence has shown that appendectomy may slightly increase the incidence of Crohn's disease [16]. In patients with acute uncomplicated appendicitis, ERAT may preserve the function of the appendix with a satisfactory therapeutic effect. To some extent, ERAT may be essential for intestinal immune function, which is another strength of the treatment.

Difficult biliary cannulation is defined as the inability to achieve selective cannulation within 10 min or 5 attempts by a recent ERCP guideline [17]. Taking the ERCP guideline as a reference, in our study, we set the difficult appendix cannulation time to within 15 min regardless of the number of cannulation attempts. In the present study, 4 patients (12.12%) failed ERAT treatment. All of these failures were due to difficult cannulation caused by a swollen mucosa and narrow lumen of the appendix. In these patients, the guidewire failed to pass through the swollen and convoluted appendiceal lumen. Unfortunately, difficult appendiceal cannulation remains an unsolved issue. We believe that there may be two ways to assist appendiceal cannulation in this situation. One is to find new equipment that can overcome the severe edema of the appendix mucosa. The other is to prolong the pre-ERAT duration to acquire more sufficient anti-inflammatory effect by antibiotics and thereby maximally reduce the inflammation of the appendiceal mucosa. However, the second method would definitely increase the overall hospitalization duration of patients. Subsequent studies will be conducted to address difficult appendiceal cannulation. In the current study, 4 patients who failed ERAT were found to have appendicoliths. However, the reason of failure was due to the mucosal edema instead of the obstruction of the appendicoliths. Apart from the 4 patients, the other 8 patients with appendicoliths had a smooth recovery by receiving ERAT alone. Hence, appendicolith should not be considered as a contraindication of ERAT. Same as previous study [18], we think the key to remove appendicoliths was based on the quality of the stone and the technique of the endoscopist. A fragile stone, regardless of its size, is more likely to be removed by the combination of irrigation and extraction basket. If the stone is hard and large, the success of retraction is more dependent on the experience and the technique of the endoscopist. When a giant and irregular appendicolith is observed, it is also feasible to squeeze the appendix and let the stone fall into the intestine cavity. After that, the appendicolith can be removed via the intestine by a stone basket. Previous studies showed that the recurrence rates of acute appendicitis patients treated with ERAT ranges from 6.1 to 15% [7–9]. In the present study, 3 patients (9.09%) had recurrent appendicitis. This recurrence rate was consistent with previous findings. The recurrence of acute appendicitis may be associated with the appendiceal morphology since long and curved appendix is also risk factor of acute appendicitis. Other factors, such as genetic and environmental factors, may also have impact on recurrence. The final crossover rate of ERAT group was 21.21%, which means more than 3/4 of all patients recovered successfully by receiving ERAT alone and those who crossed over to surgery did not experience any adverse outcomes due to the delay in appendectomy. However, the final crossover rate may be underestimated due to short-term follow-up period and a long-term follow-up is needed to be done in the future. Moreover, in a previous study, spontaneous discharge of the stents was considered as an adverse event [9]. However, in current study, the stents of 9 patients spontaneously discharged and these 9 patients did not show symptoms during the 14-day period. Thus, from our point of views, if the patients do not show any discomfort, spontaneous discharge of the stents should be considered as a benefit and convenience since these patients will not have to undergo colonoscope a second time.

Another advantage of ERAT is its ability to facilitate a precise diagnosis of acute appendicitis. Generally, endoscopy can serve as a diagnostic tool for numerous diseases, such as ulcerative colitis and Crohn’s disease [19, 20]. A previous study indicated that colonoscopy can diagnose acute appendicitis with 100% sensitivity and 99% specificity [21]. Li et al. further described the key points of diagnosing acute appendicitis by ERAT by observing both the appendiceal mucosa morphology and the appendiceal lumen imaging by fluoroscopy [8]. In our study, 33 patients with acute uncomplicated appendicitis in the ERAT group were all confirmed by ERAT. In the future, ERAT may become an essential diagnostic method for patients suspicious for acute appendicitis who have atypical clinical manifestations.

One feature of our study was that an abdominal CT scan was performed in all participants. Although CT scan is not a necessary diagnostic criterion for acute appendicitis, using CT can enhance the accuracy of diagnosing acute appendicitis and minimize the negative appendicitis rate. Similar to this view, some previous trials showed that their study design was limited due to the lack of CT scans performed in suspicious patients [7]. Based on former experiences, all participants had to be examined by abdominal CT scan in our study. Thus, we could accurately enroll the patients with acute uncomplicated appendicitis in our trial by both clinical manifestation and CT scan confirmation.

The study has several limitations. First, the success rate of ERAT largely depends on the skills of endoscopists. Although our endoscopists are experienced doctors, the lack of ERAT guidelines was still the main problem when faced with difficult cannulation. Second, since this was an RCT, we tried to perform ERAT on the first day of hospital admission, which was similar to the timing of emergency surgery. The downside of this strategy is that it may cause insufficient anti-inflammatory treatment in patients, which may increase the difficulty of appendiceal cannulation. Third, in the current study, the power was not calculated and the study is more likely to commit a type II error. However, in this pilot study, the safety and feasibility of ERAT were preliminarily estimated. Thus, future studies involved with larger sample size are needed to be done. Fourth, a double-blinded trial cannot be conducted due to the nature of the study. Last, the need to prepare for colonoscopy in patients with abdominal pain requires further study and is another limitation of the study.

In conclusion, ERAT was demonstrated to be a feasible and safe treatment for acute uncomplicated appendicitis. However, difficult appendiceal cannulation and acute appendicitis recurrence are the main problems that remain to be solved. In the future, large-scale multicenter RCTs are needed to further address the current challenges.

## Data Availability

The datasets supporting the conclusions of this article are available in the the ResMan original data sharing platform (IPD sharing platform) of the Chinese Clinical Trial Registry, which can be viewed at the following website: http://www.medresman.org.cn/pub/cn/proj/projectshow.aspx?proj=5600. We expect to release the original data on December 2022.

## Abbreviations

ERAT: Endoscopic retrograde appendicitis therapy
LA: Laparoscopic appendectomy
OA: Open appendectomy
PEG: Polyethylene glycol
SD: Standard deviation
ERA: Endoscopic retrograde appendicography
WBC: White blood cell
CRP: C-reactive protein
LOS: Length of hospital stay
VAS: Visual analog scales
ANOVA: Analysis of variance
ITT: Intention-to-treat
CT: Computed tomography
RCT: Randomized controlled trial
EMR: Endoscopic mucosal resection
